# Explosive strength and endurance adaptations in young elite soccer players during two soccer seasons

**DOI:** 10.1371/journal.pone.0171734

**Published:** 2017-02-13

**Authors:** Riccardo Di Giminiani, Christiano Visca

**Affiliations:** Laboratory of Biomechanics of the Musculoskeletal System and Motion Analysis-Department of Biotechnological and Applied Clinical Sciences, University of L’Aquila, Italy; University of Birmingham, UNITED KINGDOM

## Abstract

The purpose of the present study was to investigate the explosive strength and endurance adaptations in young elite soccer players who underwent a supervised training program for a period of two years. Nineteen players, with seven years of training experience (age: 13.3 ± 0.1 years; body weight: 57.9 ± 4.9 kg; height: 168.9 ± 4.7 cm; BMI: 20.1 ± 1.1 kg/m^2^), voluntarily participated in the present study. The testing sessions were performed at the beginning of the preparation period in the first (T1), second (T2), and third year (T3). The following performance variables were measured: explosive strength [squat-jump (SJ) and counter-movement-jump (CMJ)], pre-stretch augmentation (CMJ-SJ), leg stiffness [hopping test (HT)], short sprint performance [15 m (SSP15) and 30 m (SSP30)], aerobic endurance [test of Leger (VO2max)], maximal heart rate [at the last step of Leger (HR)], and speed-strength endurance [continuous counter-movement-jumps (CCMJ)]. A significant main effect on the VO2Max (+5.72%; F_(2.49)_ = 3.822; *p* = 0.029; ES = 1.00), HR (-1.70%; F_(2.54)_ = 3.472; *p* = 0.038; ES = 0.97), CCMJ (+7.64%; F_(2.54)_ = 5.438; p = 0.007; ES = 1.15), SJ (+10.26%; F_(2.54)_ = 15.254; p = 0.0001; ES = 1.53), CMJ (+7.36; F_(2.54)_ = 8.270; *p* = 0.001; ES = 1.33), HT (+8.34%; F_(2.48)_ = 3.297; *p* = 0.046; ES = 1.01), SSP15 (-3.50%; F_(2.44)_ = 12.760; *p* = 0.0001; ES = 1.53), and SSP30 (-4.44%; F_(2.44)_ = 5.797; *p* = 0.006; ES = 1.16) was observed in the two soccer seasons. These results highlight that, in long-term training, the monitoring of the adaptive responses in relation to the training load may provide a guideline to optimize the trainability of some performance variables in young elite soccer players (13–15 years). In the present study, we cannot exclude the influence of growth and maturation on some performance variables; therefore, the monitored adaptive responses should be considered as the possible results of an interaction between the applied training load and maturation.

## Introduction

Soccer players perform a large variety of ballistic exercises distinguished from a kinematic point of view by high velocities and accelerations throughout the entire movement [1] (i.e. jumping, kicking, sprinting, changing pace and direction) [2], that given the different situational components, are affected by the external factors such as the presence of adversaries, the characteristics of the playing surface, and the weather conditions. Therefore, the performance is affected by internal and external factors [2].

Harley et al. [3] conducted a match analysis on young soccer players (12–16 years old), investigating the various distances covered in relation to the intensity levels; the total distance covered and the distance at high (4.0–5.5 m∙s^-1^) and very high intensity (5.5–7.0 m∙s^-1^) during an official match. Their results showed that the players in the under 16 (U16) group covered greater distances than the players from U12-U13-U14 groups, in all the distances analyzed. However, the total distance covered by the young soccer players was lower than that observed in adults [4].

The several distances, covered at different intensities, are able to stimulate either the aerobic and anaerobic systems, as soccer is a sport that is characterized by intermittent periods of high speed and low speed. Elite soccer players, during the match, are able to run approximately 10 km at an average intensity close to their anaerobic threshold (80–90% of maximal heart rate) [2]. By considering the duration of the game (70–90 min) and the values of maximal oxygen consumption (VO_2_ max, 64.3 mL∙Kg^-1^ ∙min^-1^), the soccer game appears to be dependent upon the aerobic system [5]. In the latter study, the average value of the VO_2_ max in elite young players was 64.3 mL∙kg^-1^ ∙min^-1^, with a maximum value of 73.9 mL∙Kg^-1^ ∙min^-^1. Stoyer et al. [6] found that the values of VO_2_max differed in relation to the role played by the individual players (defenders/attackers/midfielders). In addition, Tomlin et al. [7] highlighted that the players with a higher VO_2_ consumption during the game also had a lower blood lactate concentration due to their higher capacity to remove the lactate during high-intensity intermittent exercise.

The average intensity in young players during a soccer match, on a regular-sized pitch, ranges from 165 to 171 bpm, about 85% of the HR max [5]. This intensity is similar to that of an adult (80–90% HR max), with the frequency values slightly higher due to a higher maximum heart rate that is age-dependent [8].

During a soccer match, young soccer players perform approximately 10–15 seconds of sprints (5, 10, and 20 m) every 90 seconds of the game (11% of the total match) [2]. Baker and Nance [9] and Comfort et al. [10, 11] found a positive correlation among sprints performance, squat jump, and maximal strength (1 RM during a back squat). Similarly, young players who trained for maximal strength (by using the leg press) improved their performance in jumping and sprinting [12, 13]. The two latter studies showed improvements in jumping that ranged from 9.4% (squat jump) to 7.4% (counter movement jump) in seventeen year-olds [12] and from 12.9% (squat jump) to 13.44% (counter movement jump) in twelve year-olds and fifteen year-olds [13] over a 8-weeks training period. In addition, the seventeen year-old soccer players increased their speed in short sprint performance by 11.9% (40 m) and the twelve and fifteen year-old reduced their sprint time by 3.3% (30 m). The greater improvements, reported by Christou et al. [13], in squat and counter movement jump, could be partially explained (as other factors could be involved) by the optimal window of trainability in twelve and fifteen year-old, which in turns depends on the stage of maturation and development of the physiological processes involved in the explosive strength. During the pubertal stage, the exponential increase in strength is accompanied by the interactive development of several factors; the nervous system, fat-free mass, theoretical fiber type differentiation, testosterone level, and biochemical characteristics [14]. Thus, strength training appears appropriate to increase the ballistic movements in skills critical in young soccer players such as jumping, turning and sprinting performance [1, 12, 13].

Bangsbo et al. [15] underline that both the aerobic and anaerobic energy systems contribute to the physiological demands of the game. The anaerobic system is considered to be of paramount importance to perform ballistic movements such as sprinting, jumping, and to change direction rapidly [2]. These actions repeated over time (speed-strength endurance) at high intensity, determines the high concentrations of lactate. In this perspective, the aerobic system plays a crucial role to increase the rate of lactate removal during the phases that are performed at lower intensities.

All the physiological parameters highlighted represent the limiting factors, and contribute towards determining the performance in young soccer players. Therefore, describing the applied training load and monitoring its impact on the physical performance may provide valuable feedback to coaches and physical trainers to optimize the development and adaptations in young soccer players. The literature lacks longitudinal investigations that have described the training effects on young soccer players, after applying a systematic load characterized by several performance variables.

### Aim

The aim of the present study was to assess the following parameters: aerobic endurance (VO_2_Max and Heart Rate), speed-strength endurance, strength, leg stiffness, and short sprint performance over two soccer seasons in young professional players (from 13 to 15 years old).

### Hypothesis

It was hypothesized that over the two years the young soccer players would improve their performance (explosive strength and endurance parameters) homogeneously.

## Materials and methods

### Study design

**Fig 1:**
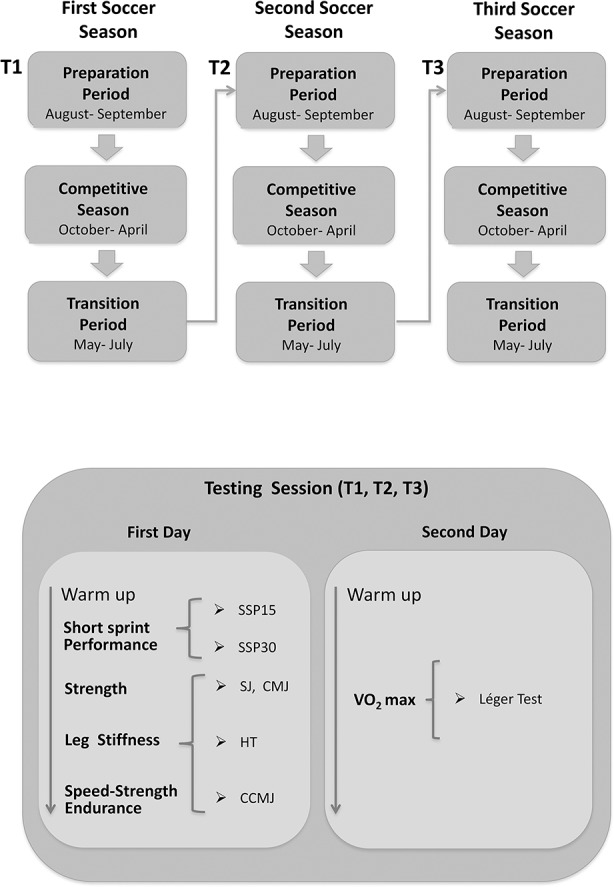
Flow diagram of the experimental design. The first and second testing sessions were performed on two different days separated by two days of rest. The measurements were taken at the same time of day for several testing days. SSP15 = Short Sprint Performance (15 m), SSP30 = Short Sprint Performance (30 m), SJ = Squat Jump CMJ = Counter Movement Jump, HT = Hopping Test, CCMJ = Continuous Counter Movement Jump.

A single-group study design with repeated measures was used. The training program was designed before the start of the first soccer season. In the first soccer season, the players participated in the “Giovanissimi National Championship” that had 28 official matches. In the second soccer season, the players participated in the “Allievi National Championship” (28 official matches). Each soccer season was divided into a preparation period, a competitive season, and a transition period (Fig 1). The testing sessions were performed at the beginning of each preparation period (Fig 1).

### Participants

Nineteen players (boys) participated in the present study. The players were thirteen years old with seven years of training experience. They did not have previous muscle, bone, or joint injuries. All the soccer players obtained a medical certification to participate in the soccer competition, and their parents provided a written informed consent before the players’ participation in the present investigation. The Ethics Committee of the Department of Biotechnological and Applied Clinical Sciences of the University approved the study. The anthropometric characteristics [average (SE)] were collected at beginning of the first (T1), the second (T2), and third (T3) soccer season (Table 1).

**Table 1:** Anthropometric characteristics of the soccer players.

Test times	Players (n°)	Age (years)	Body Weight (kg)	Height (cm)	BMI (kg/m^2^)
**T1**	19	13.2 (0.3)	57.2 (4.9)	169.5 (4.7)	19.8 (1.1)
**T2**	19	14.2 (0.3)	58.1 (3.8)	170.9 (3.0)	20.4 (1.3)
**T3**	19	15.2 (0.3)	61.8 (3.7)*†	173.9 (3.6)*†	20.8 (1.4)*

Values are means (standard error).

* Significant difference from T1 (p<0.01)

† Significant difference from T2 (p<0.05).

BMI: body mass index.

### Training

As the structure of the training program is related to the age of the players; after puberty, the periodization of training in young soccer players becomes similar to that of adults as there are windows of trainability in order to enhance the aerobic performance [16], the levels of explosive strength [17,18], short sprint performance [18], and speed-strength endurance [19,20]. The training components (modalities) were the same in the two-year program (Table 2). The preparation periods (Fig 1) of the first and second soccer season were organized in five microcycles (35 days of training) with the goal of determining the changes in all the components homogeneously, even if particular attention was given to the development of explosive strength and technical-tactical elements, mostly in the preparation period of the second soccer year (Tables 3 and 4). In fact, 110–120 min per week were dedicated to technical-tactical components and 110–130 min per week to the strength element.

**Table 2:** Training categories.

Training Categories	Aerobic Endurance	Resistance Training	Strength and Explosive Strength	Anaerobic Endurance	Technical-Tactical Exercises	Flexibility Exercises
	➢ Continuous Running ➢ Interval-Training ➢ Aerobic Shuttle-Running ➢ Small-Sides Games	➢ Push Up ➢ Pull-Ups ➢ Bench Press ➢ High and Half Squat on the Multi-power ➢ Abdominal exercise	➢ Resistance Training ➢ Short Sprint Running (Horizontal) ➢ Uphill and Downhill Sprint Running ➢ Vertical Jump ➢ (SJ, CMJ, DJ) ➢ Short Sprint on Sands on Water ➢ Hops, Multiple Hops and Bounds	➢ Shuttle Running with Change of direction ➢ Repeated Horizontal Sprint ➢ Repeated Sprints with Ball Exercises (Dribbling, Kick etc.)	➢ Coordination Drills or Group Technical Drills ➢ Technical and Tactical Exercises in Defense and Attack	➢ Static Stretching ➢ PNF ➢ Dynamic Stretching

**Table 3:** Training loads of a microcycle during the preparation period (first soccer year).

Days	Monday	Tuesday	Wednesday	Thursday	Friday	Saturday	Sunday
**Training Categories**	1 Warm-Up 2 Abdominal Exercises 3 Explosive Strength 4 Flexibility Exercises	1 Warm-Up 2 Abdominal Exercises 3 Aerobic Endurance 4 Flexibility Exercises	1 Warm-Up 2 Abdominal Exercises 3 Explosive Strength 4 Flexibility Exercises	Rest	1 Warm-Up 2 Technical-Tactical Exercises 3 Aerobic Endurance 4 Flexibility Exercises	1 Warm-Up 2 Technical-Tactical Exercises 3 Resistance Training (Upper-Body Exercises) 4 Flexibility Exercises	1 Warm-Up 2 Technical-Tactical Exercises 3 Flexibility Exercises
**Time (min)**	1 (20) 2 (10) 3 (30) 4 (10)	1 (20) 2 (10) 3 (30) 4 (10)	1 (20) 2 (10) 3 (30) 4 (10)		1 (10) 2 (35) 3 (20) 4 (10)	1 (10) 2 (25) 3 (20) 4 (10)	1 (10) 2 (50) 3 (10)

**Table 4:** Training loads of a microcycle during the preparation period (second soccer year).

Days	Monday	Tuesday	Wednesday	Thursday	Friday	Saturday	Sunday
**Training Categories**	1 Warm-Up 2 Abdominal Exercises 3 Explosive Strength 4 Flexibility Exercises	1 Warm-Up 2 Explosive Strength 3 Aerobic Endurance 4 Flexibility Exercises	1 Warm-Up 2 Abdominal Exercises 3 Technical-Tactical Exercises 4 Explosive Strength 5 Flexibility Exercises	Rest	1 Warm-Up 2 Technical-Tactical Exercises 3 Anaerobic Endurance 4 Flexibility Exercises	1 Warm-Up 2 Technical-Tactical Exercises 3 Resistance Training 4 Aerobic Endurance 5 Flexibility exercises	1 Warm-Up 2 Technical-Tactical Exercises 3 Flexibility Exercises
**Time (min)**	1 (15) 2 (20) 3 (30) 4 (15)	1 (20) 2 (20) 3 (30) 4 (10)	1 (20) 2 (10) 3 (30) 4 (10) 5 (5.0)		1 (20) 2 (25) 3 (20) 4 (15)	1 (10) 2 (15) 3 (20) 4 (30) 5 (5.0)	1 (15) 2 (55) 3 (10)

The structure of the microcycles in the preparation period was similar in both the soccer seasons (weekly days of training), whereas, the time slightly increased in the second soccer season (from 420 min to 480 min).

In the competitive season, the microcycles were organized in four-weekly sessions with the same components, but with a different proportion between the first and the second soccer season (Tables 5 and 6). The total time in the second soccer season was higher than that in the first season (380 vs. 320 min).

**Table 5:** Training loads of a microcycle during the competitive season (first year).

Days	Monday	Tuesday	Wednesday	Thursday	Friday	Saturday	Sunday
**Training Categories**	1 Warm-Up 2 Resistance Training (Upper-Body) 3 Flexibility Exercises	1 Warm-Up 2 Technical-Tactical Exercises 3 Explosive Strength or Aerobic Endurance 4 Flexibility Exercises	Rest	1 Warm-Up 2 Technical-Tactical Exercises 3 Anaerobic Endurance 4 Technical-Tactical Exercises 5 Flexibility Exercises	1 Warm-Up 2 Technical-Tactical Exercises 3 Flexibility Exercises	Rest	1 Official Match
**Time (min)**	1 (20) 2 (30) 3 (10)	1 (20) 2 (20) 3 (30) 4 (10)		1 (10) 2 (15) 3 (10) 4 (20) 5 (5.0)	1 (20) 2 (25) 3 (20)		1 (70)

**Table 6:** Training loads of a microcycle during the competitive season (second year).

Days	Monday	Tuesday	Wednesday	Thursday	Friday	Saturday	Sunday
**Training Categories**	1Warm-Up 2 Resistance Training 3 Flexibility Exercises	1 Warm-Up 2 Technical-Tactical Exercises 3 Strength 4 Aerobic Endurance 5 Flexibility Exercises	Rest	1 Warm-Up 2 Technical-Tactical Exercises 3 Anaerobic Endurance 4 Technical-Tactical Exercises or Game 5 Flexibility Exercises	1 Warm-Up 2 Technical-Tactical Exercises 3 Flexibility Exercises	Rest	1 Official Match
**Time (min)**	1 (20) 2 (30) 3 (10)	1 (10) 2 (10) 3 (25) 4 (25) 5 (10)		1 (10) 2 (10) 3 (25) 4 (30) 5 (5.0)	1 (20) 2 (50) 3 (10)		1 (80)

The training of the two transition periods (Fig 1) was not directly supervised even if a training program was given to the subjects to maintain their physical condition. The program was characterized by several alternative physical activities (i.e. jogging, beach volleyball, mountain bike, swimming, and futsal).

The training intensity in aerobic endurance was determined for each soccer player by recording the heart rate during the last step of the Léger test (this HRmax value was the maximal heart rate corresponding to the estimated VO_2_Max). Specifically, the speed at the end of the Léger test of one player was 14.5 km/h; at this speed the VO_2_max was equal to 53.9 mL · kg^-1^ · min^-1^ and the HRmax was 201 beats/min. Consequently, three training zones were defined: a) High Intensity (90–95% of the HRmax, 181–191 beats/min) and Low Duration (15 min); b) Medium Intensity (80–85% of the HRmax, 161–171 beats/min) and Medium Duration (20 min); c) Low Intensity (70–75% of the HRmax, 141–151 beats/min) and High Duration (30 min). The intensities were determined in each player. The training intensities were planned according to a linear periodization in the preparation periods (first and second year), whereas, during the two competitive seasons (first and second year) it was performed in weekly or biweekly nonlinear periodized plans [21].

The number of repetitions and sets, when explosive strength exercises were applied (Table 2), was defined on the basis of the squat jump performance. The soccer players with the highest flight times during squat jumps, performed more sets than repetitions, and vice-versa. However, the total number (numbers of repetitions × sets) was equal. This criterion is based on the subject’s fatigability and fast twitch fibers percentages [22] (subjects with the highest % fast twitch fibers in their legs develop fatigue more rapidly than the subjects with the lowest % fast twitch fibers) [23,24,25]. Therefore, to administrate explosive strength, resistance training and anaerobic endurance exercises, the soccer players were divided into three groups (low, medium, and high fatigability) and the load was applied in the following way during the first soccer year: the low group (1) performed 2 sets × 10 repetitions; the medium group (2) 3 sets × 6–7 repetitions and the high group (3) 4 sets × 5 repetitions. During the resistance training sessions the choice of the weight to be lifted was based on the individual's ability to perform “with the selected weight” the last two reps with extreme difficulty but at the same time using the correct technique (the rest period was 3 minutes between each set). During the ballistic exercises (explosive strength and short sprint performance) the players had 1 minute of rest between repetitions (i.e. short sprints) and 3–4 minutes between the sets. During the anaerobic endurance sessions, 30 seconds and 2 minutes of rest were given respectively between the repetitions and sets.

The endurance training as well as the resistance and explosive strength training were planned according to a linear periodization in the preparation periods, and in weekly or biweekly nonlinear periodized plans in the competitive seasons. However, the two nonlinear periodized strength and endurance training in the competitive seasons were out of phase.

### Testing procedures

The measurements were taken inside the sports center of the football club before starting the preparation period of the soccer seasons (Fig 1). For each soccer player, the tests were performed on two days separated by a two-day recovery period to avoid any potential fatigue effect. The measurements were taken at the same time of day in the following order: short sprint performance and vertical jumps in the first test day, rest (2 days), and Léger test on the second test day. All the data was collected in a gym with climate-controlled environment (20–21° C). Each test session began with a 20-min warm-up (6 min of treadmill running at a speed of 7 km/h; 4 min of dynamic stretching; conventional trunk, arm, and legs exercises; some vertical jumps, skip, and a brief sprint). The players were introduced to the equipment and procedures during a familiarization period on the first day. The vertical jumps (squat jump, counter movement jump, hopping, and continuous jumps) were performed on a resistive platform connected to a data collection unit (MuscleLab-Ergotest Technology, Langesund, Norway), which in turn was linked to a personal computer via a USB port.

#### Maximum oxygen consumption and heart rate

The maximum oxygen consumption (VO_2_ max) was estimated by the Léger test [26]. The test protocol consisted of a shuttle run over a distance of 20 m with incremental speeds. The starting speed was 8.5 km/h with increments of 0.5 km/h for each minute. In order for the players to maintain the pace at different speeds, a metronome with an acoustic stimulus was used. Each player changed direction by using the right and left leg alternatively to avoid fatigue of the adductor leg muscles. The test ended when the players were no longer able to follow the rhythm of the metronome (behind the line of 20 m to the acoustic signal in two consecutive shuttles). Heart rate (HR) was monitored during the execution of Léger test by using a heart rate monitor (Polar, Finland). At the end of each step, the values of the heart rates were collected. However, only the values of HR corresponding to the last speed step were used for analysis.

#### Explosive strength, pre-stretch and leg stiffness

The explosive strength was assessed by measuring the flight time during the squat jump (SJ) and the counter movement jump (CMJ). Thereafter, the rise of the center of the mass was calculated using the Bosco’ et al. formula [27]. The players performed three repetitions for each jump with their hands on their waist. The best SJ and CMJ for height were recorded for analysis. In addition, the relative change between CMJ and SJ [((CMJ-SJ)/SJ)∙100] was calculated to estimate the pre-stretch during jumping [28, 29, 30]. The leg stiffness was assessed with the hopping test (HT). The players performed a series of hops (for ten seconds) with their hands on their waists while maintaining the knee angle extended as much as possible. The players were instructed to jump as high as possible with the shortest ground contact time (maximal hopping) [31]. The consistency among the repetitions was checked by inspecting the contact time and flight time in each hop. When the contact time and flight time showed a large variability among the hops, the test was repeated (it happened in a few trials). The test was performed twice (during each testing session) with a four-min rest period between the two trials. The contact time and flight time were measured, and the leg stiffness was calculated for each hop; then, the average value across the hops was calculated [32]. The trial with the highest average value was retained for analysis.

#### Speed-strength endurance

The speed-strength endurance was assessed by the means of continuous counter movement jumps (CCMJ). In the CCMJ, the subjects were asked to perform fifteen CMJ reaching the maximum height possible in each single jump. The test was performed on a resistive platform (Ergotest Innovation, Porsgrunn, Norway) while the knee angle (at around 100 degrees) was monitored using an electrogoniometer connected to the Muscle-Lab (Ergotest Innovation, Porsgrunn, Norway).

#### Short sprint performance

The sprint time was measured using two pairs of photoelectric cells (type WL170-N132, Ergotest Innovation, Porsgrunn, Norway). The distance between the active photocell (transmitter/receiver) and the reflector was about 2 m. The distances between the two pairs of photoelectric cells were 15 (SSP15) and 30 m (SSP30) (0.8 m above the floor). The photoelectric cells were connected to the Muscle-Lab’s electronic timer (0.001 s) (Ergotest Innovation, Porsgrunn, Norway). The players started, without verbal command, from a standing position with the preferred leg behind the starting line (0.3 m). They were instructed to run as fast as possible, and for each short sprint performance distance (SSP15 and SSP30 m) two trials were performed with a four-min rest period between the trials. The best time was considered for analysis.

### Statistical analysis

When the Shapiro-Wilk’s W test revealed the non-normal distribution of the data, we applied a logarithmic transformation to obtain normal distributed responses (only one variable, pre-stretch augmentation). The untransformed data are reported (for descriptive purposes only) in the figures and expressed as mean values and standard errors (SE). The training effect on the selected variables (VO_2_ max, HR, CCMJ, SJ, CMJ, CMJ-SJ, HT, SSR15, and SSP30) was assessed over time by using one-way ANOVA repeated measures. The Bonferroni correction was used to adjust the *p*-values according to the number of comparisons that were performed. The inter-day reliability of the independent variables was quantified via the intra-class correlation coefficient (ICC, 95% confidence limit, lower confidence limit-upper confidence limit) [33]. Post-hoc analysis was executed to quantify the Cohen’s effect size (ES) for all the dependent variables. The analysis was performed using the statistical software XLSTAT 2013.2.07 (Addinsoft, SARL, New York).

## Results

The intra-class correlation coefficients (ICC) of the measured variables are reported in Table 7. The changes in height, weight and body mass index are summarized in Table 1.

**Table 7:** Reliability of the variables.

Variables	ICC	95% CL (Lower-Upper)
**VO**_**2**_**Max**	0,94	0,84–0,98
**HR**	0,80	0,42–0,94
**SJ**	0,96	0,82–0,99
**CMJ**	0,94	0,80–0,98
**CMJ−SJ**	0,53	0,03–0,84
**HT**	0,95	0,85–0,98
**CCMJ**	0,90	0,71–0,97
**SSP 15**	0,80	0,42–0,93
**SSP 30**	0,88	0,67–0,95

**ICC**: intra-class correlation coefficient, **CL**: Confidence limit

### VO_2_ max and HR

The aerobic performance improved in young elite soccer players from thirteen to fifteen years old determining a significant main effect on the VO_2_ max (F_(2.49)_ = 3.822; *p* = 0.029; ES = 1.00). The increase between T1 and T3 was significant (*p* = 0.002; ES = 1.26; +5.72%), whereas, the increase between T1 and T2 did not reach the level of significance (p = 0.080; ES = 0.81; +3.14%). Similarly, the HR decrease showed a significant main effect on the HR (F_(2.54)_ = 3.472; *p* = 0.038; ES = 0.97). Significant contrasts were found between T1 and T3 (*p* = 0.007; ES = 1.11; -1.70%), and between T2 and T3 (*p* = 0.018; ES = 1.00; -1.35%) (Fi 2).

### Speed-strength endurance (CCMJ)

**Fig 2:**
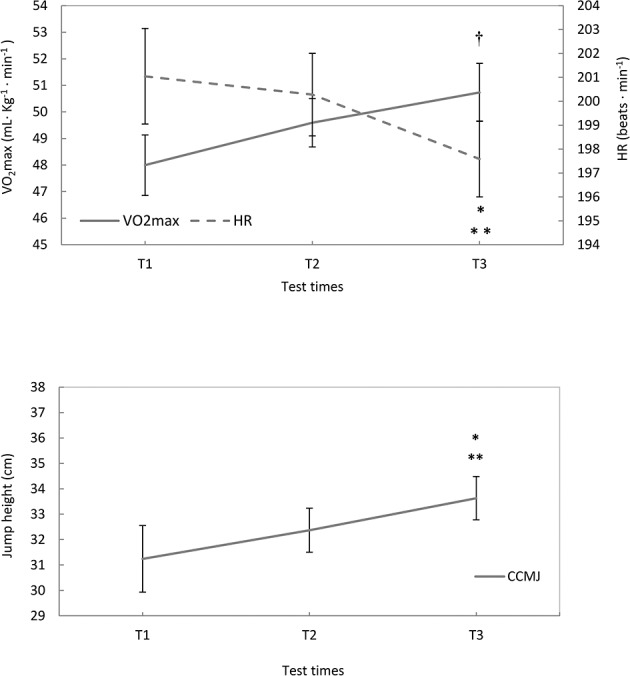
Mean values (SE) for VO_2_ max, HR and CCMJ. T1, T2 and T3 represent the test times. (VO_2_ max) ^†^Significant differences between T1 and T3 (p = 0.002). (HR) *Significant differences between T2 and T3 (p = 0.046). **Significant differences between T1 and T3 (p = 0.011). (CCMJ) *Significant differences between T1 and T3 (p = 0.0001). **Significant differences between T2 and T3 (p = 0.046).

The performance on CCMJ improved and the main effect was significant (F_(2.54)_ = 5.438; p = 0.007; ES = 1.15). The contrasts between T1 and T3 (p = 0.0001; ES = 1.62; +7.64%), and between T2 and T3 (p = 0.046; ES = 0.89; +3.9%) were significant, whereas, the difference between T1 and T2 did not reach the level of significance (p = 0.083; ES = 0.81; +3.49%) (Fig 2).

### Explosive strength (CMJ, SJ), pre-stretch (CMJ-SJ), and leg stiffness (HT)

**Fig 3:**
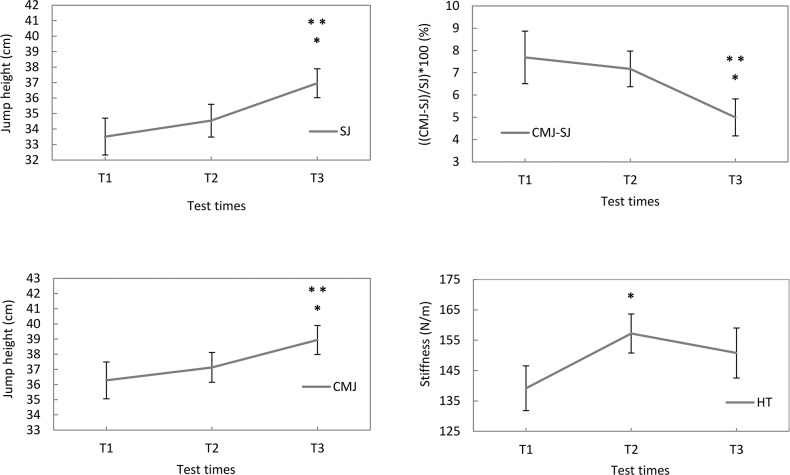
Mean values (SE) for SJ, CMJ, CMJ-SJ and HT. T1, T2 and T3 represent the test times. (SJ) *Significant differences between T2 and T3 (p = 0.001). **Significant differences between T1 and T3 (p = 0.001). (CMJ) *Significant differences between T2 and T3 (p = 0.003). **Significant differences between T1 and T3 (p = 0.0001). (CMJ-SJ) *Significant differences between T2 and T3 (p = 0.044). **Significant differences between T1 and T3 (p = 0.014). (HT) *Significant differences between T1 and T2 (p = 0.005).

The main effect over time was significant showing an increase for both SJ (F_(2.54)_ = 15.254; p = 0.0001; ES = 1.53) and CMJ (F_(2.54)_ = 8.270; *p* = 0.001; ES = 1.33). Multiple comparisons revealed significant differences between T1 and T3 (SJ, *p* = 0.0001; ES = 1.62; +10.26%) (CMJ, *p* = 0.0001; ES = 1.62; +7.36%), and between T2 and T3 (SJ, *p* = 0.0001; ES = 1.62; +6.97%) (CMJ, *p* = 0.003; ES = 1.21; +4.87%) (Fig 3).

The ratio of CMJ:SJ showed a decreasing trend over time (F_(2.50)_ = 2.575; *p* = 0.086; ES = 0.87). The differences between T1 and T3 (*p* = 0.014; ES = 1.00; -35.02%), and between T2 and T3 (*p* = 0.044; ES = 0.89; -30.40%) were both significant (Fig 3).

The main effect on HT was significant (F_(2.48)_ = 3.297; *p* = 0.046; ES = 1.01), and the contrast between T1 and T2 was significant (*p* = 0.005; ES = 1.24; +12.9%) (Fig 3).

### Short Sprint Performance (SSP15, SSP30)

**Fig 4:**
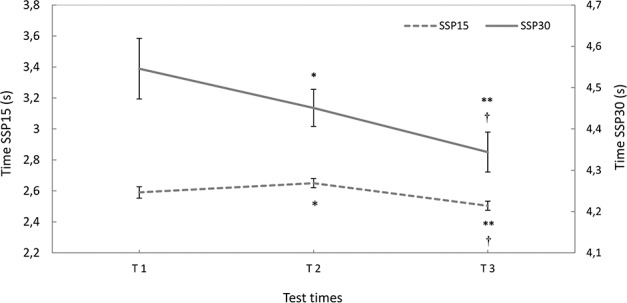
Mean values (SE) for SSP15 and SSP30. T1, T2 and T3 represent the test times. (SSP15) *Significant differences between T1 and T2 (p = 0.019). **Significant differences between T1 and T3 (p = 0.001). †Significant differences between T2 and T3 (p = 0.0001). (SSP30) *Significant differences between T1 and T2 (p = 0.0047). **Significant differences between T1 and T3 (p = 0.0001). †Significant differences between T2 and T3 (p = 0.027).

Sprint time significantly decreased and the main effects were observed on both SSP15 (F_(2.44)_ = 12.760; *p* = 0.0001; ES = 1.53) and SSP30 (F _(2.44)_ = 5.797; *p* = 0.006; ES = 1.16). The differences in SSP15 were located between T1 and T2 (*p* = 0.019; ES = 0.99; +2.31%), T2 and T3 (*p* = 0.0001; ES = 1.62; -5.51%), and T1 and T3 (*p* = 0.001; ES = 1.34; -3.32%) (Fig 4). Similarly, SSP30 showed significant differences between T1 and T2 (*p* = 0.047; ES = 0.88; -0.11%), T2 and T3 (*p* = 0.027; ES = 0.95; -2.40%), and T1 and T3 (*p* = 0.0001; ES = 1.62; -4.44%) (Fig 4).

## Discussion

The primary finding of the present study was that all the selected performance’s variables improved significantly over two-years. The variables changed linearly (increasing or decreasing) during the two-year, even though significant contrasts were achieved in the second year with the exception of the leg stiffness and short sprint performance (15m) variables. The leg stiffness variable improved only in the first year and remained constant in the second year, while the short sprint performance (15 m) variable decreased in the first year and increased in the second year. The progressive improvement in almost all the performance’s variables; particularly aerobic endurance (VO_2_ max), speed-strength endurance (CCMJ), strength (SJ and CMJ), and short sprints (SSP30), indicates that the specific training loads were well balanced to stimulate the several physiological mechanisms involved in the adaptive processes that in turn are connected to the maturation status of the young soccer players.

### Endurance adaptations

The VO_2_ max (expressed in mL ∙ kg^-1^ ∙ min^-1^) increased significantly by 5.72% after the two-year soccer seasons (approximately by 3% during each year) (Fig 2). In the training schedule, several methods were used to determine a large effect size (ES>0.80) in the aerobic endurance (Table 3). Additionally, the intensity was individualized according to the maximal heart rate recorded during the last step of the Léger’ test (see training load in the “methods” section).

Unfortunately, we cannot make direct comparisons of our results with those of others because longitudinal studies with a similar duration, protocol, and age of the subjects are lacking in the literature. In the most comprehensive mixed longitudinal study that was conducted by Baxter-Jones et al. [16] in young soccer players (13–15 years old), the VO_2_ max remained stable from the pre-pubertal stage to the mid-pubertal stage (55.7 mL ∙ kg ∙ min^-1^), but increased significantly from the mid-to-late pubertal stage (14–15 years old, from 55.7 to 61.5 mL ∙ kg^-1^ ∙ min^-1^, +10.4%). Conversely, Berg et al. [34] did not report significant changes in the VO_2_ max and HR max. However, the soccer players in the study were younger (~12 years old) and the training period was shorter (9-wks). These latter studies support the view that the adolescent’s growth is the critical period to develop the VO_2_ max by the means of a “specific training” as it showed a decline in the growth period of a 13–15 year old, gymnasts, and untrained individuals [16]. In this context, we noted that the training in our study was more effective in the second year (from 14 to 15 years), along with a concomitant increase in the training volume (one session bi-weekly in the first competitive season and one session weekly in the second competitive season). An annual training structure similar to our study (including one high-intensity training session for VO_2_ max weekly during a competitive season) did not significantly change the VO_2_ max of young soccer players (16–17 years old) [35]. A higher level of aerobic endurance has been reported in the McMillan et al. study [36], which showed that high intensity aerobic interval training sessions (90–95% of maximal heart rate) twice per week for ten weeks in addition to normal soccer training, increased the VO_2_ max from 63.4 to 69.8 mL ∙ kg^-1^ ∙ min^-1^ (+ 10%) in young elite soccer players (17 years old). Our opinion is that a direct comparison with the latter investigations cannot be made because of the different experimental designs of the studies in question. Specifically, our investigation represents a “systematic monitoring" of the performance over two years that has taken into account different performance variables that could be influenced by several factors (i.e. training, competitions, growth and maturation). On the other hand, a specific study was analyzed [36] that showed meaningful results in a "relatively short-term period". In other words, our study has been carried out with a broader perspective than that of the above studies which have focused on a limiting factor only and/or short-term training.

Therefore, future investigations should assess the gain in performance when additional specific short-term training, similar to that of MacMillan et al. [36], is integrated in a long-term training process (i.e. two years) with other variables.

We have to underline that in our study the VO2Max was (not directly measured) estimated by means of the Legér test, therefore, the endurance performance, that is; the distance covered during the test represent a global measure that could also include adaptations of the anaerobic system, improvements in running economy, and the anaerobic threshold [5]. In reality, it is necessary to add that in the monitoring of the aerobic endurance in the young players, direct methods, measuring the oxygen consumption with an oro-nasal mask, are unlikely used for several practical reasons (long time test runs and high costs).

The anaerobic system was stimulated with specific speed-strength endurance exercises that significantly increased the performance during continuous counter movement jumps (CCMJ; +7.7% overall and +3.9% in the second year). This test is able to grasp specific adaptions in the anaerobic system as the counter-movement jumps maximally performed for fifteen seconds are sustained by the ATP-PCr capacity maximally, in addition to the maximal glycolytic power [37]. Although, half of the total time (fifteen seconds) was spent in the air, the remaining half of the contact time was used to store elastic energy for reuse in the next positive phase of the jump [23, 28], as trained adolescents (12–15 years) have less glycolytic ability than adults [19, 20]. Therefore, the duration of fifteen seconds seems appropriate for adolescents given their limited functionality of the glycolytic system, to highlight adaptations in speed-strength endurance associated with the recruitment of fast motor units [23].

The decrease in the maximal heart rate associated with an increase in the distance during the test of Leger, cannot be explained only with the growth, as on an average the maximum heart rate decreases by 1 beat/min per year [38]. However, in our study, the heart rate decreased by approximately 5 beats/min from 13 to 15 years. The decrease of the maximal heart rate associated with the growth and the aerobic training could indicate an optimization of the heart rate-stroke volume relationship to maximize the cardiac output [39]. In a recent work of Vesterinen et al. [40], the heart rate-running sprint index has been proposed as a method for monitoring the adaptation to endurance training in running without the need to use a maximal running test. The heart rate-running sprint seems to be significantly related to maximal oxygen uptake and to the changes of peak running speed [40].

### Explosive strength adaptations

The effective improvements in the aerobic performance were obtained with no concomitant negative interference effects on the neuromuscular system (explosive strength and sprinting performance). The explosive strength (CMJ and SJ) started improving in the first soccer season by 2.49 and 3.29%, respectively. However, the improvements were markedly higher at the end of the second soccer season (by 4.87 and 6.97%, respectively). These large improvements, in the second soccer season, could be explained by the increase of the training load performed by the players in the preparation periods (two sessions weekly vs. three sessions weekly) and during the competitive seasons (1 session bi-weekly vs. 1 session weekly) and/or by the maturation status. Similar results have been reported in the longitudinal studies conducted on young elite English [18] and Serb soccer players [17], in which the jump performance during a CMJ increased by about 1.25% (30), and by 3.51% [18] from thirteen to fourteen years of age, and by 5.14% [18] from fourteen to fifteen years of age. However, in the latter investigations, detailed training content in order to provide valuable feedback and information concerning the dose-response relationship were not reported.

In specific short-term training, the highest improvement in jump performance (CMJ, +14.4%) by elite young soccer players (14.5 yr.) have been shown in a study by Buchheit et al. [41], where one session of strength was added per week during the ten weeks of normal training regimen. Makhlouf et al [42] found that by combining the same training sequence as our study (strength before endurance within a single training session in young elite players under the age of 14 years) without including the exercises with external load, the squat jump (+10.5%) and counter movement jump (+7.3%) improved after a 12-week period in which two sessions per week were included in the training regimen of the competitive season. Marques et al. [43] found that by superimposing a combined plyometric and sprinting regimen (two sessions of 20 minutes per week for six consecutive weeks) to the normal training soccer (four sessions per week) in young elite players (13.4 yrs.), jump performance increased by 7.7% in a short period of time (6-weeks).

Similarly to the endurance training, these specific latter studies demonstrate that effective improvements in explosive strength can be obtained in young elite soccer players by increasing the workload when the training focuses only on one exercise type and is performed in a short-term period. As explained above, the main issue is optimizing the dose-response relationship when more exercise types (explosive strength, endurance, and technical-tactical exercises) are involved in the training over a longer period of time (soccer season). In addition, during concurrent training, the antagonistic intracellular signaling mechanisms could determine the inhibition of strength improvements (inhibition of muscle hypertrophy) when the strength and endurance variables are stimulated simultaneously in a training schedule [44, 45].

An interesting feature of the present study was that the pre-stretch had a tendency to decrease from the first (-4.6%) to the second year (-30.4%) while the leg stiffness increased during the first year (+12.9%) and then had a tendency to slightly decrease over the second year. The opposite changes of the pre-stretch augmentation and leg stiffness in the first soccer season are in line with the inverse relationship between the pre-stretch augmentation and the musculotendinous stiffness [30] or tendon stiffness [29]. In our study, the decrease in the pre-stretch cannot be explained by the growth of the players as with development (from 4 to 20 years old) pre-stretch increases [28]. However, we estimated the leg stiffness [32] by using a multi-joint exercise (hopping); therefore, the results cannot be directly compared with other studies in which the stiffness was determined in different ways (tendon or musculotendinous stiffness). In this context, the tendon stiffness only accounted for 22% of the variance in the pre-stretch augmentation [29]. Thus, other factors such as the potentiation in the activation level of muscles and the re-utilization of elastic energy stored in cross-bridges could be the potential mechanisms responsible for the different adaptive responses between the pre-stretch augmentation and leg stiffness from the first to the second soccer season.

Recently, Ramirez-Campillo et al. [46, 47, 48] have shown large improvements in reactive strength index in drop jump (from 12 to 36%) and short sprint performance 10–30 m (from -0.4 to -6.0%) during a biweekly short-term (i.e., 6–7 weeks) plyometric training interventions in young soccer players (10–15 years), with no background in regular strength or plyometric training. The concomitant improvements in the reactive strength index (or leg stiffness during hopping) and short sprint performance are not surprising as hopping in place has a basic mechanical feature similar to the spring-mass model used during running [49]. In fact, Chelly and Denis [49] have evidenced a significant correlation between the leg stiffness during hopping in place and the maximal velocity during sprint performance (40 m). On the contrary, the initial acceleration phase is dependent on the muscle strength and power. In this connection, Chelly et al. [12] have shown that by applying a resistance training program with heavy loads that is performed twice a week for two months in young soccer players (17 years old), the relative change of one repetition maximum at back squat (+25%) and the speed in the first phase of short sprint performance (+25%) was higher than the relative change in squat jump (+10%) and maximal speed running of 40 m (+12%).

To summarize, these studies explain the improvements seen in SSP30 with an increase in the leg stiffness (unlike SSP15) in the first soccer season of our study. Conversely, in the second year when the improvements in the SJ and CMJ became more pronounced, significant increases were also achieved in SSP15.

### Limitations

In the present investigation there was no control group to balance the experimental one, therefore we cannot differentiate between adaptations due to training or growth and maturation status. In general, the physical performance could be influenced by the related processes of growth and maturation. Therefore, the growth of young players from 13 to 15 years, who have experienced significant changes in height, weight and body mass index, may have influenced the performance’s variables selected in the present study. In the literature, several standardized tests; such as sprints, shuttle runs and vertical jumps showed improvements, on average, from childhood through adolescence in boys; even if tasks in which the body is projected, (jumps and short sprints) correlate negatively with body mass [50]. Similarly, the dependence of aerobic power on body size during growth has been indicated in the growth curve of relative aerobic power (i.e., per unit body mass, mL·kg^-1^ ·min^-1^); the values in longitudinal studies show a decline through adolescence VO_2_max per unit body mass (mLO_2_ · kg^-1^ · min^-1^), generally begins to decline one year before PHV (peak height velocity) and continues to decline after PHV [50].

In synthesis, the advantages for explosive strength adaptations with maturation, that are related to the interactive development of the following factors: the nervous system, fat-free mass, theoretical fiber type differentiation, testosterone level, and biochemical characteristics [14], could be counteracted by the somatic growth in strength performance characterized by the action of the human body against the gravity [50]. Consequently, in the present study we cannot ascertain the relative role of training, growth and maturation on the adaptive process of some variables (i.e. explosive strength, speed-strength endurance, short sprint performance, leg stiffness and heart rate).

## Conclusions

Firstly, the tests used in the present study are practical and reliable predictors to monitor explosive strength, and endurance performance changes in young elite soccer players. Secondly, the training structure and the improvements evidenced provide helpful guidelines of expected longitudinal gains in endurance and strength performance of elite soccer players from 13 to 15 years.

## Supporting information

S1 File Dataset. (PDF)
