# Supplementary material for: Explosive strength and endurance adaptations in young elite soccer players during two soccer seasons
Source: PLoS One. 2017 Feb 13;12(2):e0171734. doi: 10.1371/journal.pone.0171734 (PMC5305282; doi:10.1371/journal.pone.0171734)
Supplement: S1 File — (PDF) [file pone.0171734.s001.pdf]

Subjects (ID)	Time	HR	SP15	SP30	SJ	CMJ	CMJ-SJ	HT	VO2max	CCMJ
1	t1	209	2,726	4,969				65,4	50,4	
1	t2	208	2,736	4,569	31,4	34,8	9,7	114,2	50,7	31,547
1	t3	196	2,514	4,449	36,4	36,8	1,2	150,2	57,3	32,007
2	t1				25,5	27,4	7,45	136,6		22,907
2	t2	205	2,84	4,757	28,5	31,4	9,4	140	51	26,7
2	t3	189	2,55	4,561	33,1	34,1	2,9	155,7	41,9	28,153
3	t1	201	2,792	4,612	25	31,6	20,9	119	40,2	22,273
3	t2	203	2,817	4,676	26,9	29,7	9,6	197,2	44	26,32
3	t3	208	2,491	4,401	32,8	33,8	3	129,1	51	28,78
4	t1	187	2,721	4,623	37,4	40,9	8,6	136,3	41,1	32,173
4	t2	184	2,617	4,413	36,1	39,3	8,2	153,8	45,6	35,687
4	t3	181	2,629	4,558	31,8	36,9	13,6	191,6	44,7	32,16
5	t1	196	2,668	4,862	33,5	34,3	2,3		45,5	27,927
5	t2				33,7	36	6,2			
5	t3	198	2,576	4,412	35,2	35,8	1,7		44,3	
6	t1	214	2,373	4,261	41,4	46,3	10,5	168,7	54,8	42,273
6	t2	200	2,538	4,228	41,3	41,8	1,2	175	51,6	36,587
6	t3	208	2,332	4,162	43,7	45,6	4,1	189,7	56,2	34,513
7	t1	198	2,377	4,255	39,6	41,7	5,1	174,2	51	33,58
7	t2	203	2,499	4,284	37,8	42,3	10,7	181,8	47,4	32,453
7	t3	195	2,387	4,192	40	42,8	6,6	149,2	49,2	33,027
8	t1		2,724	4,85	27,9	29	3,9		44	28,513
8	t2									
8	t3	196	2,484	4,363					51,6	
9	t1	205	2,49	4,692	37,3	40,8	8,6	154,8	56,2	40,8
9	t2	201	2,705	4,507	38,5	39,7	2,9	145	54,5	37,621
9	t3	201	2,591	4,406	40,9	44	7,1	123,4	55,9	38,22
10	t1		2,683	4,056	30,4	31,4	3,2	108,5	47,4	27,113
10	t2				31,5	34,7	9,3	128,8		33,253
10	t3	190	2,659	4,598	32,1	32,9	2,2	82	51,3	30,22
11	t1	193	2,56	4,408	29,3	35,4	17,3	161,1	52,2	
11	t2	202	2,675	4,587	32,5	36,9	11,9	169,6	51	29,793
11	t3	198	2,649	4,529	35,1	36,9	4,9	158,3	44,7	34,82
12	t1	202	2,412	4,265	35,8	37,1	3,4	133,5	46,8	32,587
12	t2	200	2,452	4,284	37,8	40,2	6	162,6	46,8	34,173
12	t3	199	2,526	4,274	40,6	41,2	1,5	133,3	50,4	36,593
13	t1	212	2,795	4,859	33,9	36,5	6,9	141,8	47,7	27,847
13	t2	206			32,6	36,2	10	151,2		31,147
13	t3	202			35,1	35,9	2,3	124,6	50,1	28,853
14	t1	202	2,365	4,101	34,8	35,8	2,7	109,8	40,8	34,333
14	t2		2,493	4,279	37,2	37,4	0,6	142,5	44	33,533
14	t3		2,292	4,021	38,4	38,9	1,3	162,5	47,1	35,693
15	t1				36,1	40,3	10,5	146,7		36,04

15	t2	210	2,484	4,106	40,8	41,6	2	182,3	57,9	38,407
15	t3	203	2,351	3,968	40,2	43,3	7,2	171,8	57,9	39,9
16	t1				27,1	30,2	10,2	194,9		26,787
16	t2	192	2,666	4,458	31,7	35,6	11	213	49,2	28,521
16	t3	193	2,437	4,382	33,7	37,4	9,8	234	49,8	31,7
17	t1	188			35,6	39,4	9,7	128,2	51	
17	t2	190	2,609	4,478				125,8	51,3	
17	t3	197	2,46	4,278	39,8	42,2	5,6	141,8	54,5	37,507
18	t1	216	2,564	4,432	41,4	42,2	1,7	121,3	46,2	34,753
18	t2	209	2,506	4,166	40,2	43,4	7,2	152,5	52,8	35,18
18	t3	207	2,317	4,05	44,8	46,4	3,5	132,1	55,1	39,593
19	t1	198	2,755	5,014	29,7	31,5	5,6	165,4	53,4	28,76
19	t2				27,8	29,6	6,3	136,9		28,54
19	t3	195	2,691	4,422	33,4	37,5	11,1	134,3	51	34,32
